# Levels II and III neck dissection for larynx cancer with N0 neck

**DOI:** 10.5935/1808-8694.20120009

**Published:** 2015-11-20

**Authors:** Carlos Takahiro Chone, Hugo Fontana Kohler, Rodrigo Magalhães, Marcos Navarro, Albina Altemani, Agricio Nubiato Crespo

**Affiliations:** a MD, PhD (Professor, Department of Otolaryngology Head and Neck, University of Campinas); b MD (Department of Otolaryngology Head and Neck, University of Campinas); c MD, PhD (Professor, Department of Surgical Pathology, University of Campinas); d MD, PhD (Professor, Department of Otolaryngology Head and Neck, University of Campinas). Department Otolaryngology Head and Neck, University of Campinas, Brazil

**Keywords:** laryngeal neoplasms, lymph nodes, lymphatic metastasis, neck dissection

## Abstract

The removal of level II, III, and IV metastases has gained importance in the treatment of squamous cell carcinomas (SCC) of the neck and larynx. This study assessed the possibility of removing level II and level III metastases only, given the low likelihood of occurrence of metastatic lymph nodes on level IV in SCCs of the larynx.

**Objective:**

This study aimed to analyze the prevalence rates of metastatic lymph nodes on level IV in laryngeal SCC patients.

**Methods:**

This prospective study enrolled consecutive patients with laryngeal SCC submitted to neck lymph node dissection. Neck levels were identified and marked for future histopathology testing.

**Results:**

Six percent (3/54) of the necks had level IV metastatic lymph nodes. All cN0 necks (42) were free from level IV metastasis. Histopathology testing done in the cN (+) necks (12) revealed that 25% of the level IV specimens were positive for SCC. The difference between cN0 and cN (+) necks was statistically significant (*p* = 0.009). Level IV metastases never occurred in isolation, and were always associated with level II or level III involvement (*p* = 0.002).

**Conclusion:**

The prevalence rate for lymph node metastasis in cN0 necks was 0%. Level IV metastatic lymph nodes were correlated to cN (+) necks. Level IV metastasis was associated with the presence of metastatic lymph nodes in levels II or III.

## INTRODUCTION

The proposal of radical neck dissection for treatment of patients with head and neck squamous cell carcinoma (HNSCC) began with Crile in 1906^1^. Its routine use declined with time and modified procedures gained wider popularity. Based in the predictable pattern of neck metastasis for HNSCC^2^^,^^3^, selective neck dissection was developed, with dissection of most affected levels of neck. If these nodes bear no metastatic disease, the incidence of metastases in other levels is low and its elective removal is of questionable value.

Regarding laryngeal cancer, a study of 262 radical neck dissections^2^^,^^3^, observed that metastatic disease occurred at levels II to IV, with sporadic metastasis to level I or V. Based on this findings, they proposed as elective treatment for these patients the dissection of levels II to IV. In a study of 384 neck dissections by serial sections^4^, the main sites of metastatic disease were levels II-IV. The occurrence of metastatic disease in levels I or V was associated usually with enlarged nodes at levels II to IV^2^^,^^3^. Furthermore, the Brazilian Head and Neck Cancer Study Group^5^ demonstrated no difference in outcome between patients submitted to a modified neck dissection or a lateral neck dissection in the elective treatment of patients with laryngeal cancer. Nowadays, dissection of levels II to IV is considered the treatment of choice for N0 laryngeal neoplasms^6^ because the low probability of metastases to level V ranging between 0% and 7% in glottic SCC and 5% to 9% for supraglottic SCC^1^, ^2^, ^3^, ^4^, ^5^^,^^7^, ^8^, ^9^, ^10^, ^11^.

The aim of this study was to determine the incidence of level IV metastasis in patients with laryngeal squamous cell carcinoma (SCC) correlating it to clinical nodal status of neck.

## METHOD

Consecutive patients with laryngeal SCC were prospectively evaluated between 2007 and 2011. For each patient, demographic, clinical and pathological data were recorded. Clinical and pathological staging was recorded according to 2006 AJCC staging system^12^. The local Institu tional Review Board approved the study protocol under code CAAE 0020.0.146.000-07. Informed consent was obtained from all patients submitted to this study. All patients had histopathological confirmation of SCC. All patients were staged at neck and primary site with computer tomography scan before treatment. Only patients submitted to surgical treatment with simultaneous neck dissection were included. Patients with cN0 necks with supraglottic cancer were submitted to bilateral neck dissection in stages T1 to T4, but in glottic cancer through T3, with dissection of levels II to IV, ipsilaterally. In cases with cN (+) necks it was performed modified or radical neck dissection through levels I to V and contralateral dissection of levels II, III and IV. Each level was removed in a separate fashion, respecting the surgical landmarks intraoperatively, unless in cN2a disease, where some levels was put together as the mestastasis was removed in block and detachment of levels means disruption of mass and in this case the levels was noted together.

The surgical specimen of neck levels was evaluated by conventional hystopathological analysis with hematoxilin and esosin (HE). It was obtained three slices from each lymph node for HE standard evaluation, one from the middle of lymph node and other from the center of each half of original lymph node.

Patients with previous treatment, pharyngeal extension of lesion or N3 necks or without inclusion criteria were excluded from the study. Patients submitted to non-surgical treatment were also excluded from the study.

In presence of positive margins or extracapsular spread of lymphatic metastases, patient was submitted to adjuvant chemoradiotherapy with cisplatin at the dose of 100 mg per square meter of body surface on day 1, 22 and 43 intravenously with conventional fractionation of 2 Gy per fraction per day to 66 Gy of treatment. Otherwise, patients without the features but with perineural invasion, angiolymphatic embolus, multiple metastases, stage III or IV disease were submitted to adjuvant radiation therapy of 60 Gy in 30 fractions. Patient not submitted to postoperative adjuvant treatment were excluded of study.

It was compared the rate of metastatic disease between cN (0) and cN (+) necks.

Statistical analysis was performed using the Fisher test with statistical significance at 0.05 level.

## RESULTS

Thirty one patients met the inclusion and exclusion criteria. There were 26 males and five females, with a median age of 64 years at the time of diagnosis. Fifteen patients presented advanced glottic cancer and 16 supraglottic cancers. The distribution of theses tumors by clinical and pathological stage was shown in Table 1. Twenty-five were submitted to total laryngectomy (all T3 and T4 lesions) and six to partial horizontal laryngectomy(T2 supraglottic cancer). Twenty-three patients were submitted to bilateral neck dissection and eight to unilateral neck dissection. Bilateral neck dissection was done in 16 supraglottic ones, four of these with clinically and radiologically positive unilateral necks, and in seven glottic cases with positive necks. No bilateral positive necks were observed on clinical and radiological evaluation.

**Table 1:** Distribution of patients according to staging.

Clinical tumor staging				
Glottic cancer				
T1	T2	T3	T4	
–	5	10	–	
			Total	15
Supraglottic câncer				
T1	T2	T3	T4	
–	–	7	9	
			Total	16
Pathological tumor staging				
T1	T2	T3	T4	
0	3	7	21	
			Total	31
Clinical neck staging				
cN0	cN1	cN2a	cN3	
20	6	5	–	
			Total	31
Pathological neck staging				
pN0	pN1	pN2a	pN3	
15	11	5	–	
			Total	31

Twenty patients were cN (0) and eleven had palpable lymph nodes only at the same side of the lesion cN (+). Patients were staged as cN0 in 20 patients, cN1 in six patients(level II) and cN2a in five patients(levels II and III).

Fifty-four neck dissections were obtained. There were 43 cN0 neck dissections and eleven cN+ neck dissections. Of selective neck dissections, 34 to 47 lymph nodes were harvested from surgical specimen (mean = 39) and from radical or modified neck dissections 61 to 84 lymph nodes (mean = 67).

No patients staged as cN0 presented histopathological metastasis at level IV. Among those patients with clinically positive neck nodes, there was a 25% (3/11) incidence of metastasis to level IV at histopathological evaluation. There was a significant difference in rate of metastases at level IV among patients cN0 and cN+ (*p* = 0.009). No patient presented isolated metastasis to level IV. There was a significant difference in the compromise of level IV when levels II or III were also positive on pathological evaluation (*p* = 0.02). Five neck specimens (12%) that were clinically negative converted to pathologically positive, only at level II, without metastases to level IV. Of eleven clinically positive necks, all were pathologically positive on levels II or III.

## DISCUSSION

The routine inclusion of level IV in elective neck dissection for laryngeal SCC has been questioned due to the low incidence of metastasis and the potential complications associated with this procedure, particularly on left side due to thoracic duct.

Tu^13^ studied 142 patients with supraglottic laryngeal SCC submitted to dissection of levels II and III with no pathologically neck metastases. The neck dissection specimens of 142 patients were negative for metastasis. Fifteen of the 142 patients (10.6%) had neck recurrences during the follow-up of 5 years. The recurrence rate of this series with limited dissection on the neck was comparable with those reported in the literature after radical or modified neck dissection^14^. In a recent study, the rate of occult positive lymph node metastasis at level IV, with no evidence of disease at levels II and III was 1.4%, in patients with endolaryngeal SCC^15^.

Ambrosch et al.^16^ published a study with 503 patients with HNSCC treated with elective dissection including only levels II and III and recurrences were not observed at level IV with a mean follow-up of 41 months. In this same study, level IV was dissected only in patients with suspicious or pathologically positive (by frozen section analysis) nodes at level II or III. The authors conclude that elective dissection of level IV is not necessary in oropharyngeal, laryngeal and hypopharyngeal SCC.

León et al.^8^ in a study with similar design included 79 patients with endolaryngeal SCC. Neck dissection included only levels II and III with frozen section analysis of these levels and level IV and V were removed only when positive nodes at levels II or III were found. After a minimum follow-up of 24 months, there were no regional recurrences in this series without dissection of level IV. Khafif et al.^11^ studied 71 patients with larynx cancer submitted to elective neck dissection. The rate of metastases on level IV was 2.6%, with no differences between transglottic and supraglottic carcinomas. Among the patients with clinically enlarged lymph nodes, the rate of level IV compromise was 32% in the ipsilateral neck and 0% at the contralateral side. Other study, collected the data of three prospective multi institutional trial. Pathologic and molecular studies of neck dissection specimens in 175 patients with larynx cancer with cN0 necks found only 3,4% of occult metastases in level IV^17^.

## CONCLUSION

The results of these studies support that dissection of level IV is not mandatory in patients with cN0 laryngeal SCC.

The results of our study agree with those previously presented. The rate of metastasis to level IV was 0% in cN (0) necks despite 12% were histopathologically positive in level II, but without metastases at level IV and its dissection should not be considered in the cN0 neck.

## References

[1] Byers RM, Wolf PF, Ballantyne AJ. (1988). Rationale for elective modified neck dissection. Head Neck Surg..

[2] Candela CC, Shah J, Jaques DP, Shah JP. (1990). Patterns of cervical node metastases from squamous carcinoma of the larynx. Arch Otolaryngol Head Neck Surg..

[3] Shah JP. (1990). Patterns of cervical lymph node metastasis from squamous carcinomas of the upper aerodigestive tract. Am J Surg..

[4] Lindberg R. (1972). Distribution of cervical lymph node metastases from squamous cell carcinoma of the upper respiratory and digestive tracts. Cancer..

[5] End results of a prospective trial on elective lateral neck dissection vs type III modified radical neck dissection in the management of supraglottic and transglottic carcinomas. Brazilian Head and Neck Cancer Study Group. Head Neck. 1999;21(8):694-702. 10.1002/(sici)1097-0347(199912)21:8<694::aid-hed3>3.0.co;2-b 10562681

[6] Ferlito A, Silver CE, Rinaldo A, Smith RV. (2000). Surgical treatment of the neck in cancer of the larynx. ORL J Otorhinolaryngol Relat Spec..

[7] Yang CY, Andersen PE, Everts EC, Cohen JI. (1998). Nodal disease in purely glottic carcinoma: is elective neck treatment worthwhile?. Laryngoscope..

[8] León X, Quer M, Orús C, Sancho FJ, Bagué S, Burgués J. (2001). Selective dissection of levels II-III with intraoperative control of the upper and middle jugular nodes: a therapeutic option for the N0 neck. Head Neck..

[9] Redaelli de Zinis LO, Nicolai P, Tomenzoli D, Ghizzardi D, Trimarchi M, Cappiello J (2002). The distribution of lymph node metastases in supraglottic squamous cell carcinoma: therapeutic implications. Head Neck..

[10] Spriano G, Piantanida R, Pellini R, Muscatello L. (2003). Elective treatment of the neck in squamous cell carcinoma of the larynx: clinical experience. Head Neck..

[11] Khafif A, Fliss DM, Gil Z, Medina JE. (2004). Routine inclusion of level IV in neck dissection for squamous cell carcinoma of the larynx: is it justified?. Head Neck..

[12] American Joint Committee on Cancer (2006). Cancer staging Handbook from the AJCC Cancer Staging Manual.

[13] Tu GY. (1999). Upper neck (level II) dissection for No neck supraglottic carcinoma. Laryngoscope..

[14] Strong EW. (1969). Preoperative radiation and radical neck dissection. Surg Clin North Am..

[15] Lim YC, Choi EC, Lee JS, Koo BS, Song MH, Shin HA. (2006). Is dissection of level IV absolutely necessary in elective lateral neck dissection for clinically N0 laryngeal carcinoma?. Oral Oncol..

[16] Ambrosch P, Kron M, Pradier O, Steiner W. (2001). Efficacy of selective neck dissection: a review of 503 cases of elective and therapeutic treatment of the neck in squamous cell carcinoma of the upper aerodigestive tract. Otolaryngol Head Neck Surg..

[17] Ferlito A, Silver CE, Rinaldo A. (2008). Selective neck dissection (IIA, III): a rational replacement for complete functional neck dissection in patients with N0 supraglottic and glottic squamous carcinoma. Laryngoscope..

